# LncRNA profile study reveals a seven-lncRNA signature predicts the prognosis of patients with colorectal cancer

**DOI:** 10.1186/s40364-020-00187-3

**Published:** 2020-02-28

**Authors:** Rui Huang, Lian Zhou, Yue Chi, Haibo Wu, Lei Shi

**Affiliations:** 1 School of Life Sciences, Chongqing University, Chongqing, 400044 People’s Republic of China; 2 Chongqing University Cancer Hospital, Chongqing, 400044 People’s Republic of China

**Keywords:** Colorectal cancer, LncRNA, Prognosis, Risk score, Biomarker

## Abstract

**Background:**

The prognosis of colorectal cancer (CRC) is still challenging to evaluate or predict. Recently, long non-coding RNAs (lncRNAs) have been found to play an important role in tumorigenesis and prognosis, however, few lncRNAs have been identified in CRC progression. We aimed to establish a lncRNA signature to improve prognosis prediction of CRC.

**Methods:**

In the present study, we profiled lncRNA expression with a lncRNA-mining approach in two CRC data sets from Gene Expression Ominus (GEO) (GSE39582, *N* = 557 and GSE17538, *N* = 200). LncRNAs were analyzed to determine a prognostic signature by Cox regression and Robust likelihood-based survival model. We identified seven lncRNAs that significantly associated with the disease free survival (DFS) in the training group. A risk score formula was constructed to evaluate the performance of this lncRNA panel.

**Results:**

A seven-lncRNA signature was established to predict prognosis of CRC patients. The prognostic value of this signature was verified in the training group, internal validation group and external validation cohort, respectively. Receiver operating characteristic (ROC) analysis suggested a powerful discrimination ability of the seven-gene signature. Finally, Cox regression analyzed this signature as an independent influencing factor and subsequent pathway or network analysis implicated a potential mechanism of these lncRNAs.

**Conclusions:**

In summary, the seven-lncRNA signature we identified can effectively classify patients. This risk score model could serve as an independent biomarker to predict prognosis of CRC patients.

## Background

Colorectal cancer (CRC) is one of the leading causes of cancer death. CRC alone accounts for more than 10% of all cancer cases worldwide, and is a heavy burden on human life and economy [1, 2]. It has been estimated that in total 1,800,977 cases occurred and 861,663 people died in 2018 [2]. The current standard treatment of CRC, which has significantly improved overall survival (OS), includes surgery followed by adjuvant chemotherapy and in some cases in combination with targeted biologics. However, treatment outcome still remains undesirable. Histological diagnosis has shown valuable but insufficient prediction for prognosis of CRC patients. An increasing evidence proposes that the discovery and development of molecular biomarkers will accelerate the identification of potential high risk CRC patients and their prognostic evaluation.

In the last ten years, genomic approaches were used to facilitate the systematic analysis of changes in RNA and protein expression associated with disease diagnosis and outcome [3]. Long non-coding RNAs (lncRNAs) are newly discovered non-coding RNAs, which have received considerable attention recently in human cancers [4–6]. LncRNAs are defined by no less than 200 nucleotides in length that lack significant protein-coding capability [7]. Despite of this, lncRNAs are believed to play an important role in regulating gene expression, contribute to multiple biological processes [8, 9]. A growing number of lncRNAs are found to be intimately associated with prognosis of patients such as HOTAIR in lung cancer [10], DANCR in hepatocellular carcinoma [11] and MALAT1 in different cancer types [12]. A growing number of evidence suggests that the aberrant expressions of lncRNAs have been associated with CRC [13]. According to a recent study on lncRNA RP11, upregulated expression of RP11 was associated with increased CRC risk and high possibility of metastasis [14]. However, the research of prognosis-related lncRNA in CRC has not been extensively investigated. Therefore, establishing a prognostic lncRNA signature might be a promising strategy for the prognosis prediction of CRC patients.

One vital challenge in searching prognostic lncRNAs is the availability of publicly available data sets, which should contain both lncRNA profiles and clinical prognostic information. RNA-seq is an extensive way to profile lncRNA expression. However, since the small sample size and the restricted access of raw data, applicable RNA-seq data sets of CRC are relatively limited. In contrast, there are a larger number of microarray profiles, including hundreds of CRC samples with clinical information. For example, 585 samples were included in the GSE39582 data set and 557 of them have disease free survival (DFS) time and status. In addition, MMR (mismatch repair), adjuvant chemotherapy, KRAS mutation and seven more clinical variables were included in this data set [15]. Moreover, microarray based expression profiling may have better sensitivity for low-abundance transcripts [16], which could benefit relative low expressed lncRNA screening [17]. Although these original arrays are not designed for lncRNA profiling, previous studies have indicated that lncRNAs can be interrogated by mining the microarray raw data [18–20].

In the present study, we applied this method to re-annotate gene expression of lncRNAs on a data set of 557 patients from GSE39582, as well as another independent GSE17538 cohort. By using the sample-splitting method, Cox regression analysis and Robust likelihood-based survival modeling, we identified a prognostic, seven-lncRNA signature to evaluate the risk score from the GSE39582 training group patients, and validated it in the internal GSE39582 validation group and another independent external GSE17538 cohort. Patients with high risk score have relatively poor prognoses than those with low risk score, in both training and validation datasets.

## Materials and methods

### CRC data sets

The purpose of this study was to identify a signature of lncRNAs that can be served as an effective prognostic marker for CRC patients. Data sets and corresponding clinical data were downloaded from the publicly available Gene expression Omnibus (GEO, NCBI, http://www.ncbi.nlm.nih.gov/geo/) [21]. Two large cohorts of CRC microarray data from the Affymetrix Human Genome U133 plus 2.0 platform were included in this study: GSE39582 [15] and GSE17538 [22]. There were 585 and 244 CRC patients, respectively. The CRC samples in GSE39582 were randomly split into a training group (*N* = 279) and an internal validation group (*N* = 278). Moreover, the CRC samples in GSE17538 were analyzed as an external validation cohort.

### Data analysis of microarrays

Raw microarray data were downloaded as CEL files from GEO and analyzed using “Oligo” package from R software. All analyses were performed as standard instructions and summarized briefly. Firstly, raw data were checked for quality to exclude any experimental artifacts. Then each microarray data set was normalized individually using Guanine Cytosine Robust Multi-Array Average method (GCRMA) [23]. After background correction and normalization, expression values represented by multiple probes (or probe sets) were collapsed by taking the mean value of the set of probes. All the expression data and sample phenotypes were prepared for subsequent analysis.

### LncRNA profile annotation

LncRNA profiling on the Affymetrix-based GEO data sets was achieved by a well-established mining method [20]. Briefly, the information for each lncRNA, incluing Ensembl ID, Ensembl transcript ID and symbol, was downloaded from the GENCODE database (release 19). Meanwhile, the Ensembl transcript ID and the RefSeq ID for lncRNAs were downloaded from the HGNC database. Finally, the symbols and RNA types for each probe were obtained by matching the two datasets. Probes matched more than one lncRNAs were discarded. For multiple probes matching one lncRNA, gene expression was summarized by computing the mean value of the probes to represent the expression level of single lncRNA.

### Conduction of the risk formula for prognostic prediction

The risk score formula was constructed using the GSE39582 training group (*N* = 279). Firstly, by performing univariate Cox proportional hazards regression analysis with R package “survival”, the association between the lncRNA expression and patient’s DFS was assessed. LncRNA with a parametric *P* value of less than 0.01 was included in the subsequent analysis. Secondly, these significant lncRNAs were further evaluated with a permutation test using Biometric Research Branch-Array (BRB-Array) Tools, which calculated a permutation *P* value for each lncRNA based on 10,000 random permutations [24]. LncRNA with a permutation *p* values of less than 0.01 was considered statistically significant. Next, lncRNAs that passed the above criteria were employed for subsequent analysis with robust likelihood-based survival modeling, by using “rbsurv” R package [25]. The parameters involved were set as default except for the maximum number of gene, which was set as 20. To construct a predictive model, the selected lncRNAs were fitted into a multivariable Cox regression model in the training group as described [26, 27]. Then a risk formula was established based on a linear combination of the expression level of these lncRNAs, weighted by their regression coefficients derived from the multivariate Cox regression model [26, 27]. Finally, risk score was computed for each patient with this formula and patients were classified into high risk or low risk group, by taking the median risk score as a cutoff point. By using R package “survminer”, Kaplan-Meier estimate was assessed to compare the survival difference between the high risk and low risk groups in each data set. The significance was calculated with the log-rank test and set at 0.05. To test whether the risk score was independent of clinical variables, multivariable Cox regression and stratification analysis were performed. All statistical analyses were carried out with the Bioconductor [28] and R Version 3.5.1 (R Development Core Team 2018). Significance levels for *P* values were set at 0.05 unless indicated.

### ROC curve

Receiver operating characteristic (ROC) curves were employed to compare the sensitivity and specificity of the survival prediction based on the risk score model. Time-dependent ROC of the risk score were analyzed by “tdROC” R package and visualized with “ggplot2” package.

### Gene set enrichment analysis (GSEA)

GSEA is a powerful computational algorithm that determines whether a pre-defined set of genes shows differences between two groups [29]. GSEA was performed with the JAVA program (http://software.broadinstitute.org/gsea/index.jsp) against MSigDB C2 Reactome gene sets as described previously [30]. Genes were ranked with the metric of absolute “signal to noise” value and 1000 random sample permutations were carried out.

### LncRNAs interaction networks

Proteins and miRNAs interacted with these seven lncRNAs were searched in ENCORI, previously starBase v3.0 (The Encyclopedia of RNA Interactomes, http://starbase.sysu.edu.cn/index.php) with default parameters [31]. Total 55 proteins were identified associated with 6 lncRNAs and 87 miRNAs were interacted with 3 lncRNAs. The networks were visualized with Cytoscape software (v3.7.2) [32].

## Results

### Data sets characteristics

The following two large cohorts of CRC microarray data obtained from GEO were included in this study: GSE39582 [15] and GSE17538 [22]. There were 585 and 244 CRC patients, respectively. After removal of samples without DFS data, each of 557 and 200 patients were included in our analysis. For Kaplan-Meier analysis, samples were filtered according to the corresponding clinical data, as shown in each figure. Samples in GSE39582 were randomly split into a training group (*N* = 279) and an internal validation group (*N* = 278). In addition, the CRC samples in GSE17538 served as an external validation data set. Additional file 1: Figure S1 summarizes the work flow of the entire experiment.

### Identification of seven lncRNAs for prognosis prediction in the training group

After re-annotation, we got 3783 affymetrix probes for HGU133 plus 2.0 microarray. For each data set, 3005 unique lncRNAs were included in our study after standard data processing procedure. The training data set was used for the identification of prognostic lncRNA genes. Univariable Cox regression analysis was performed and a total of 104 lncRNAs correlated with DFS, whose parameter *P*-values were less than 0.01, were chosen for next analysis. By subjecting the 104 lncRNAs to permutation test using BRB-Array tools, we narrowed down this panel to 93 lncRNAs with permutation P-value < 0.01. Those 93 lncRNAs were further analyzed by Robust likelihood-based survival modeling [25]. This algorithm selects survival-associated genes based on the partial likelihood of the Cox model and discover multiple sets of genes by iterative forward selection [25]. Using this method, seven lncRNAs were screened out as the predictor signature and their detailed information were shown in Table 1. Of these, positive coefficients for the six genes (CTD-2354A18.1, NR2F1-AS1, AC073283.1, MIR31HG, AL132709.8, RP11-834C11.4) indicated that their upregulated levels of expression were associated with shorter survival. The negative coefficient indicated that upregulated level of expression of AC069278.4 was associated with longer survival, suggesting that it may be a tumor suppressor gene.

**Table 1:** Seven lncRNAs significantly associated with the disease free survival in the training group patients (*N =* 279)

Ensembl ID	Gene symbol	Permutation *P* value^a,b^	Hazard ratio^a^	Coefficient^a^	Diseases^c^
ENSG00000261780	CTD-2354A18.1	1.00E-07	5.743	1.75	Pathogenesis of gastric cancer, Overall survival of colorectal cancer
ENSG00000237187	NR2F1-AS1	1.00E-07	4.451	1.49	Multiple cancer
ENSG00000225187	AC073283.1	4.00E-04	3.269	1.18	NA
ENSG00000171889	MIR31HG	1.00E-07	2.064	0.72	Senescence, Osteogenesis of adipose stem cells, Progression of multiple cancer
ENSG00000288302	AL132709.8	1.00E-07	1.951	0.67	Ovarian cancer
ENSG00000250742	RP11-834C11.4	2.00E-04	1.769	0.57	NA
ENSG00000267242	AC069278.4	1.00E-07	0.174	−1.75	NA

Abbreviations: *NA* Not Available

^a^ Derived from the univariable Cox proportional hazards regression analysis in the 279 training group patients

^b^ Obtained from permutation test repeated 10,000 times

^c^ Detailed in discussion section

### The seven lncRNA-based risk score model and the survival in the training group

**Fig. 1:**
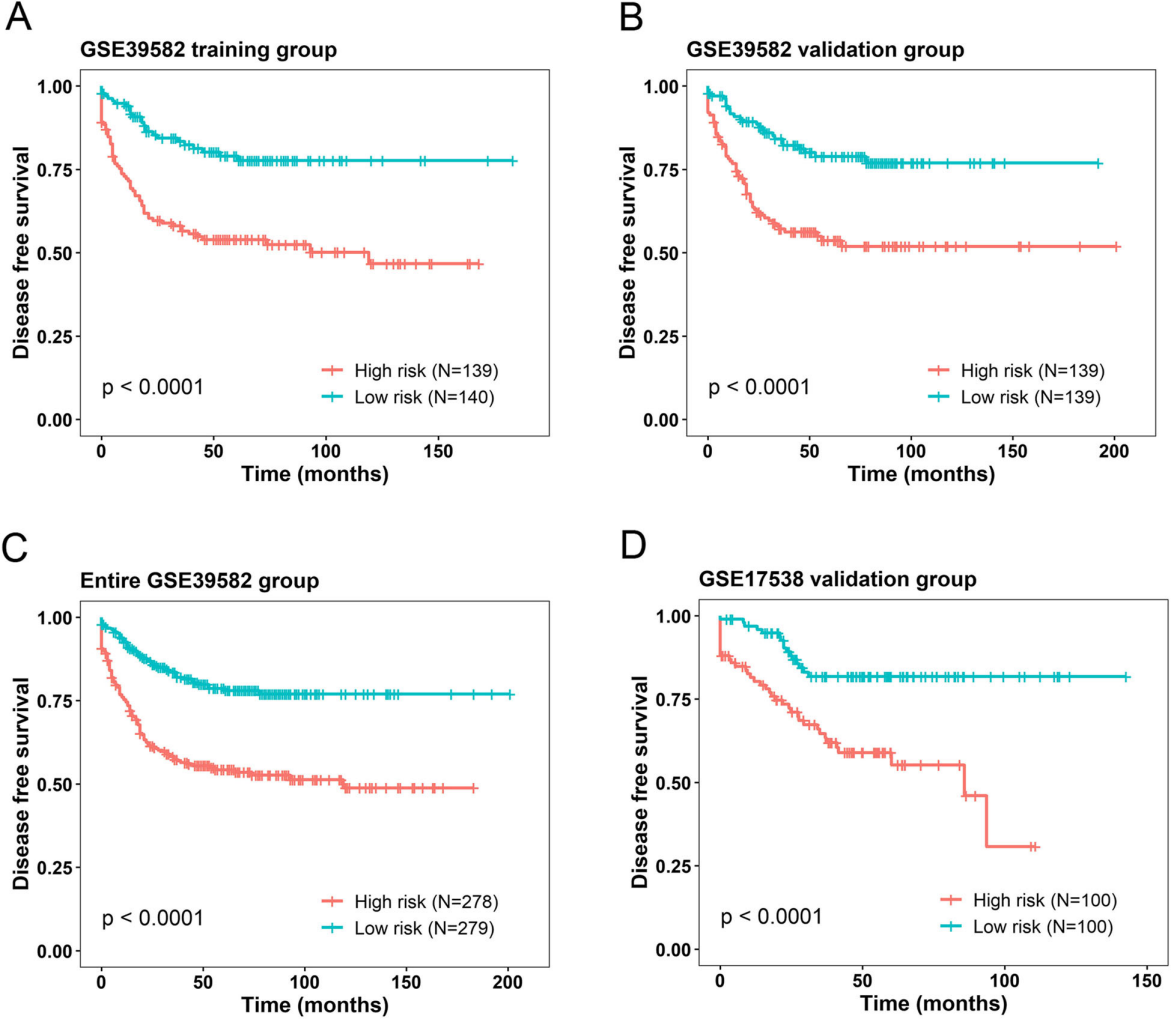
Kaplan-Meier analysis (estimates) of the disease free survival (DFS) of GEO datasets using the seven-lncRNA signature. The Kaplan-Meier plots were used to visualize the DFS probabilities for the patients based on the risk score evaluation. **a** Kaplan–Meier curves for GSE39582 training-group patients (*N* = 279); **b** Kaplan–Meier curves for GSE39582 internal validation-group patients (*N* = 278); **c** Kaplan–Meier curves for the entire GSE39582 patients (combined training and validation group patients, *N* = 557). **d** Kaplan–Meier curves for the external validation GSE19538 patients (*N* = 200). The tick marks on the curves represent the censored events. The differences between the two curves were determined by the two-sided log-rank test

To integrate all these seven lncRNAs identified in our previous step, we performed a Cox multivariable regression analysis on the training group. A prognostic model based on the coefficients was developed and the risk score formula was constructed as the following: risk score = (0.852 × the expression level of *CTD-2354A18.1*) + (0.674 × the expression level of *NR2F1-AS1*) + (0.848 × the expression level of *AC073283.1*) + (0.193 × the expression level of *MIR31HG*) + (0.034 × the expression level of *AL132709.8*) + (0.264× the expression level of *RP11-834C11.4*) + (− 1.226× the expression level of *AC069278.4*). We then calculated the seven-lncRNA signature risk score of each patient in training group using the above formula. The median risk score (5.760) was used as the cutoff point to divide the training set into two groups, high risk (*N* = 139) and low risk groups (*N* = 140). We evaluated the DFS, showing the survival time of high risk group is significantly shorter than the low risk group (log-rank test *P* < 0.0001) (Fig. 1a). The association of the seven-lncRNA risk score with DFS was also significant when it was evaluated as a continuous factor in both univariable and multivariable Cox regression model (*P* = 6.03E-14 and *P* = 1.82E-12, respectively) (Table 2).

**Table 2:** Univariable and multivariable Cox regression analyses in each data set

Variables	Univariable model^a^			Multivariable model^a^		
	HR	95% CI of HR	*P* value^b^	HR	95% CI of HR	*P* value^b^
GSE39582 training (*N* = 278)						
Seven-lncRNA risk score	2.71	2.087–3.519	< 0.001	2.749	2.111–3.579	< 0.001
Age	0.999	0.983–1.014	0.877	1.01	0.993–1.027	0.261
Gender	1.457	0.951–2.233	0.084	1.428	0.925–2.205	0.108
TNM stage I	1.00(referent)			1.00(referent)		
TNM stage II	10,245,861	0.000-	0.994	5,864,621	0.000-	0.995
TNM stage III	16,355,014	0.000-	0.993	7,823,016	0.000-	0.994
TNM stage IV	52,014,059	0.000-	0.993	42,817,026	0.000-	0.994
GSE39582 validation (*N* = 274)						
Seven-lncRNA risk score	1.978	1.558–2.511	< 0.001	1.875	1.435–2.449	< 0.001
Age	1	0.984–1.016	0.977	0.998	0.982–1.015	0.839
Gender	1.117	0.73–1.709	0.609	1.559	0.996–2.442	0.052
TNM stage I	1.00(referent)			1.00(referent)		
TNM stage II	4.42	0.598–32.673	0.145	3.223	0.434–23.944	0.253
TNM stage III	10.644	1.465–77.354	0.02	5.997	0.808–44.512	0.08
TNM stage IV	48.847	6.533–365.212	< 0.001	40.881	5.415–308.664	< 0.001
Entire GSE39582 (*N* = 552)						
Seven-lncRNA risk score	2.263	1.907–2.685	< 0.001	2.248	1.879–2.69	< 0.001
Age	0.999	0.988–1.011	0.927	1.004	0.992–1.015	0.538
Gender	1.278	0.946–1.726	0.110	1.536	1.132–2.084	0.006
TNM stage I	1.00(referent)			1.00(referent)		
TNM stage II	7.479	1.036–53.984	0.046	4.652	0.642–33.689	0.128
TNM stage III	14.37	1.999–103.274	0.008	6.9	0.95–50.115	0.056
TNM stage IV	55.189	7.558–402.996	< 0.001	42.134	5.746–308.933	< 0.001
GSE17538 validation (*N* = 200)						
Seven-lncRNA risk score	1.945	1.317–2.873	< 0.001	1.905	1.206–3.008	0.0057
Age	0.98	0.962–0.999	0.043	0.989	0.968–1.01	0.312
Gender	0.75	0.441–1.276	0.289	0.848	0.481–1.493	0.567
TNM stage I	1.00(referent)			1.00(referent)		
TNM stage II	5.177	0.668–40.132	0.116	4.313	0.552–33.69	0.163
TNM stage III	10.278	1.387–76.172	0.023	8.064	1.076–60.457	0.0423
TNM stage IV	49.435	6.615–369.41	< 0.001	40.052	5.299–302.762	< 0.001

Abbreviations: *HR* hazard ratio, *CI* confidence interval

^a^ In both univariable and multivariable Cox regression analyses, risk score and age were evaluated as continuous variables, and gender and TNM stage were evaluated as category variables

^b^*P* < 0.05 was considered statistically significant in all analyses

### The prognostic values of seven-lncRNA signature in validation groups

In order to confirm our findings, we used two validation groups to test the above signature. The corresponding risk scores were calculated according to the risk formula. Patients in GSE39582 internal validation set were classified into a high risk (*N* = 139) and a low risk group (*N* = 139) using the median score (5.721) of the validation set as the cutoff point. Consistent with the findings described above, patients in high risk group showed significantly shorter DFS than patients in low risk group (log-rank test *P* < 0.0001) (Fig. 1b). Similar results were also observed for the entire GSE39582 data set (cut point 5.742) (Fig. 1c). For further verification, external validation set GSE17538 was employed and patients in high risk group (*N* = 100) showed shorter DFS than patients in low risk group (cut point 10.04, log-rank test *P* < 0.0001) (Fig. 1d). In the Cox regression model, in which the seven-lncRNA risk score was evaluated as a continuous variable, similar correlation could be achieved (Table 2).

### Risk score distribution and ROC analysis

**Fig. 2:**
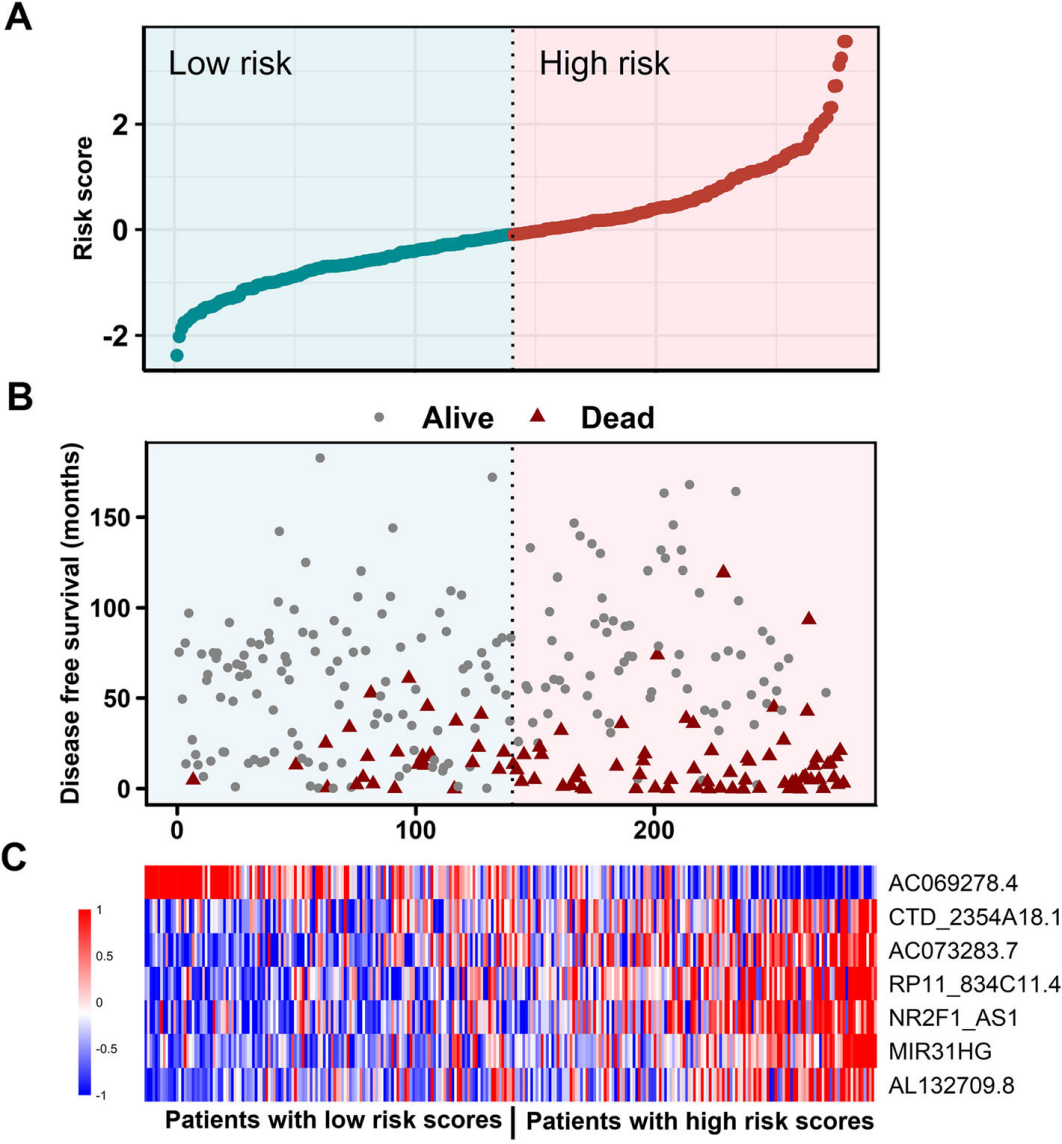
LncRNA risk score performance in the GSE39582 training dataset. The distribution of signature risk score, patients’ survival status and seven-lncRNA expression were analyzed in the GSE39582 training patients (N = 279). **a** The distribution of lncRNA signature risk score distribution; **b** The survival status and time of corresponding patient; **c** The heatmap of the lncRNA expression value. Rows represent lncRNAs and columns for patients. The black line means the median risk score cutoff dividing patients into low-risk and high-risk groups

We also visualized risk score distribution in these data sets. The samples were ranked according to their risk scores (Fig. 2a) and survival status of patients were showed as in Fig. 2b. A heatmap was visualized to demonstrate the expression profiles of these seven-lncRNAs (Fig. 2c). We found that patients with low risk scores tended to express high levels of protective lncRNAs (*AC069278.4*), whereas patients with high risk scores show a preference for high levels of the other six lncRNAs. Similar results can be observed for internal validation group, entire GSE39582 data set, and external GSE17538 cohort (Additional file 1: Figure S2-S4).

**Fig. 3:**
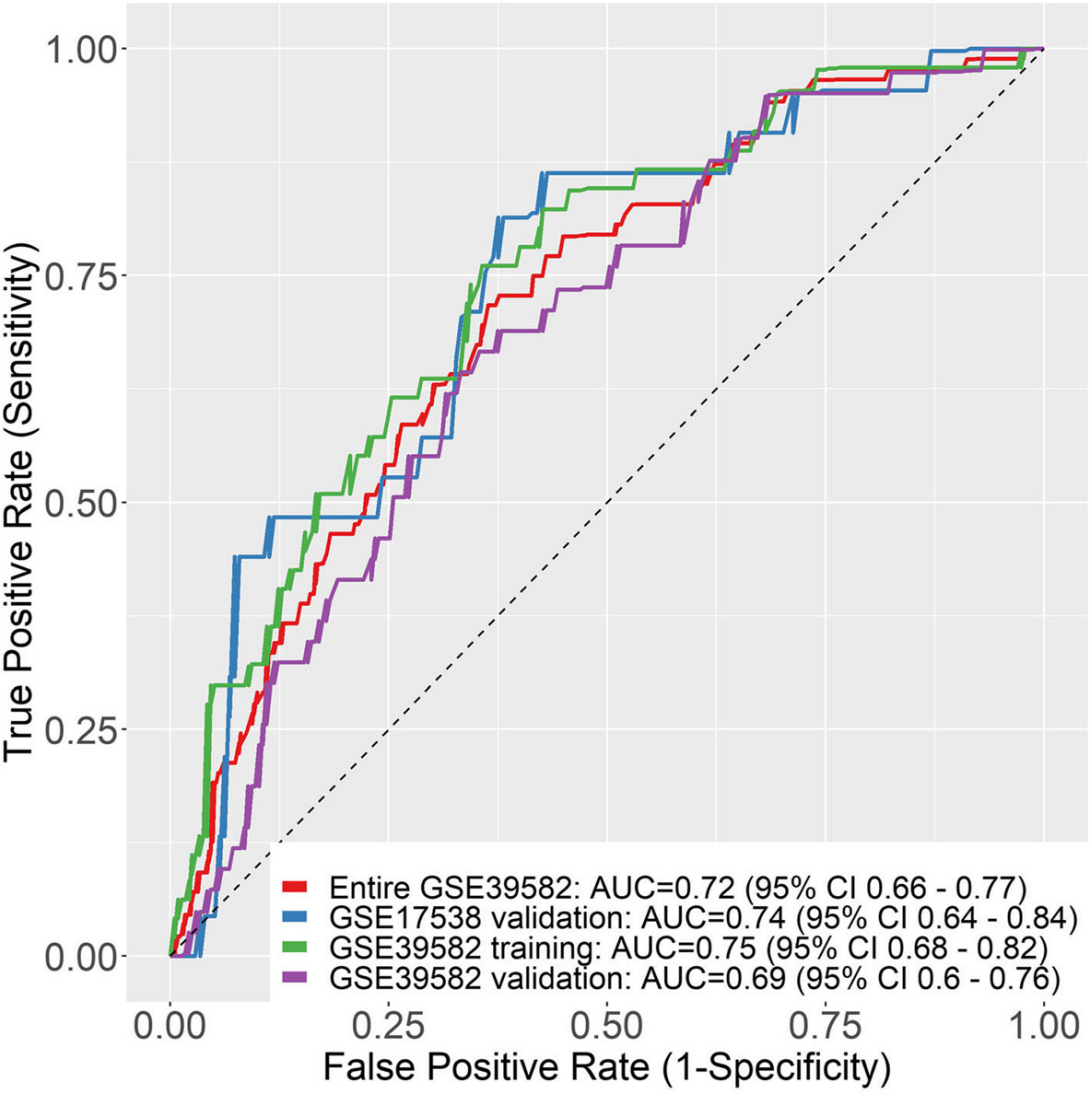
ROC curves of seven-lncRNA risk score in different datasets. The receiving operating characteristic curve (ROC) of risk score were calculated for GSE39582 training group (green), GSE39582 validation-group (red) entire GSE39582 (purple) and entire GSE39582 (blue)

To further investigate the discrimination power of the signature, ROC curves based on the calculated risk score were created within each inspected data set. The area under the curve (AUC) of GSE39582 training group was 0.75 (95% CI, 0.68–0.82), showing a strong separation ability (Fig. 3). In addition, the AUCs were 0.69 (95% CI, 0.6–0.76), 0.72 (95% CI, 0.66–0.77), 0.74 (95% CI, 0.64–0.84) for internal validation, entire GSE39582 and external GSE17538 validation data sets, respectively (Fig. 3). We can learn from this analysis, take in whole, that our seven-lncRNA signature had a strong prognostic value.

### The prognostic values of seven-lncRNA signature is independent of TNM stage

**Fig. 4:**
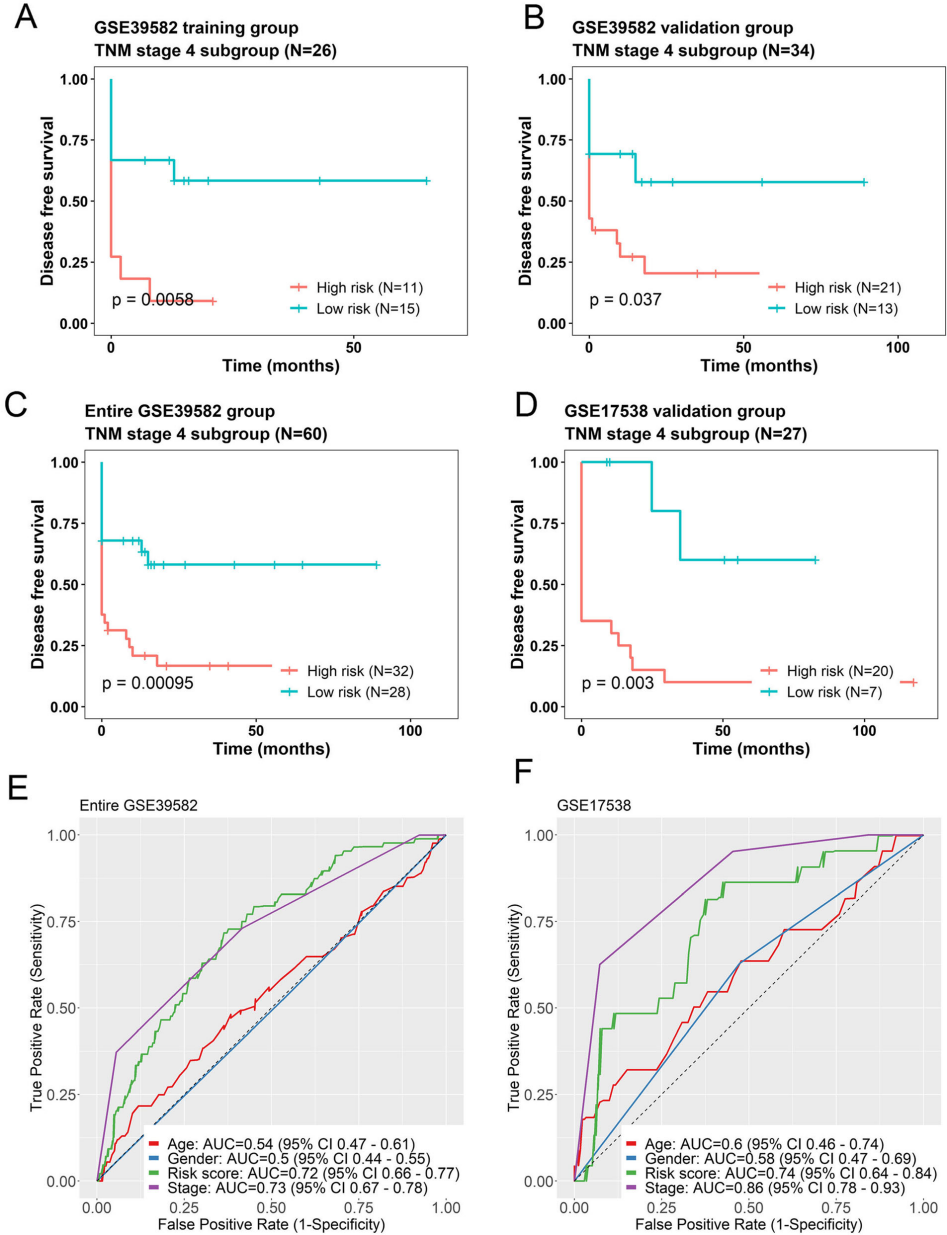
The seven-lncRNA signature was associated with prognosis in TNM stage 4 patients. Kaplan-Meier analysis of the disease free survival (DFS) of patients with stage 4 in training group (**a**), internal validation group (**b**), entire GSE39582 dataset (**c**) and GSE19538 validation dataset (**d**). The ROC curves of seven-lncRNA risk score (green), age (red), gender (blue) and stage (purple) were shown in entire GSE39582 (**e**) and GSE17538 (**f**), respectively

To further investigate the prognostic values of the seven-lncRNA signature, Cox regression analyses were performed based on the clinical characteristics, including age, gender, TNM stage in all cohorts (Table 2). Our analysis demonstrated that the seven-lncRNA risk score remained to be significantly associated with DFS when adjusted by other variables in every group. According to TNM stage system for CRC, patients were divided into four subgroups (I, II, III and IV). Data stratification analysis was then conducted and showed that the seven-lncRNA signature had the ability of predicting prognosis in stage IV only. Kaplan–Meier curves for the high and low risk groups in stage IV patients were drawn. The results suggested that patients with high risk scores exhibited poorer DFS than those with low risk scores. Above observations were conducted in the training group (Fig. 4a, log-rank test *P* = 0.0058), the internal validation group (Fig. 4b, log-rank test *P* = 0.037), entire GSE39582 (Fig. 4c, log-rank test *P* < 0.001) and the GSE17538 validation group (Fig. 4d, log-rank test *P* = 0.003).

We also performed ROC analysis to identify the sensitivity and specificity of survival prediction of the seven-lncRNA risk score, age, gender and stage on these patients. As shown in Fig. 4e, the AUC for age and gender are comparatively low (0.54 and 0.5, respectively) in GSE39582 data set. When compared with TNM stage, the AUC of the seven-lncRNA risk score was much the same (0.72 versus 0.73). Similarly, the AUC results had almost a same pattern like GSE39582 (Fig. 4f). The AUC for age and gender were low (0.6 and 0.58), and comparatively high for risk score and stage (0.74 versus 0.86). These analyses indicated that seven-lncRNA signature may have a better predictive ability than age and gender, and have an equivalent predictive power with TNM stage IV. Taking together, the seven-lncRNA signature was more favorable in our analysis.

### The prognostic values of seven-lncRNA signature is independent of adjuvant chemotherapy

Furthermore, we wanted to know whether the prognostic value of the seven-lncRNA signature was independent of all other clinical characteristics. There was adjuvant chemotherapy, KRAS mutation, mismatch repair (MMR) status and 5 more clinicopathological factors in GSE39582 data set. The corresponding sample number and univariable Cox analysis for each factor were shown in Additional file 2: Table S1. Unfortunately, of the 244 patients from GSE17538, no additional such clinical information was available for the patients, so the following results were only evaluated in GSE39582 data set.

**Fig. 5:**
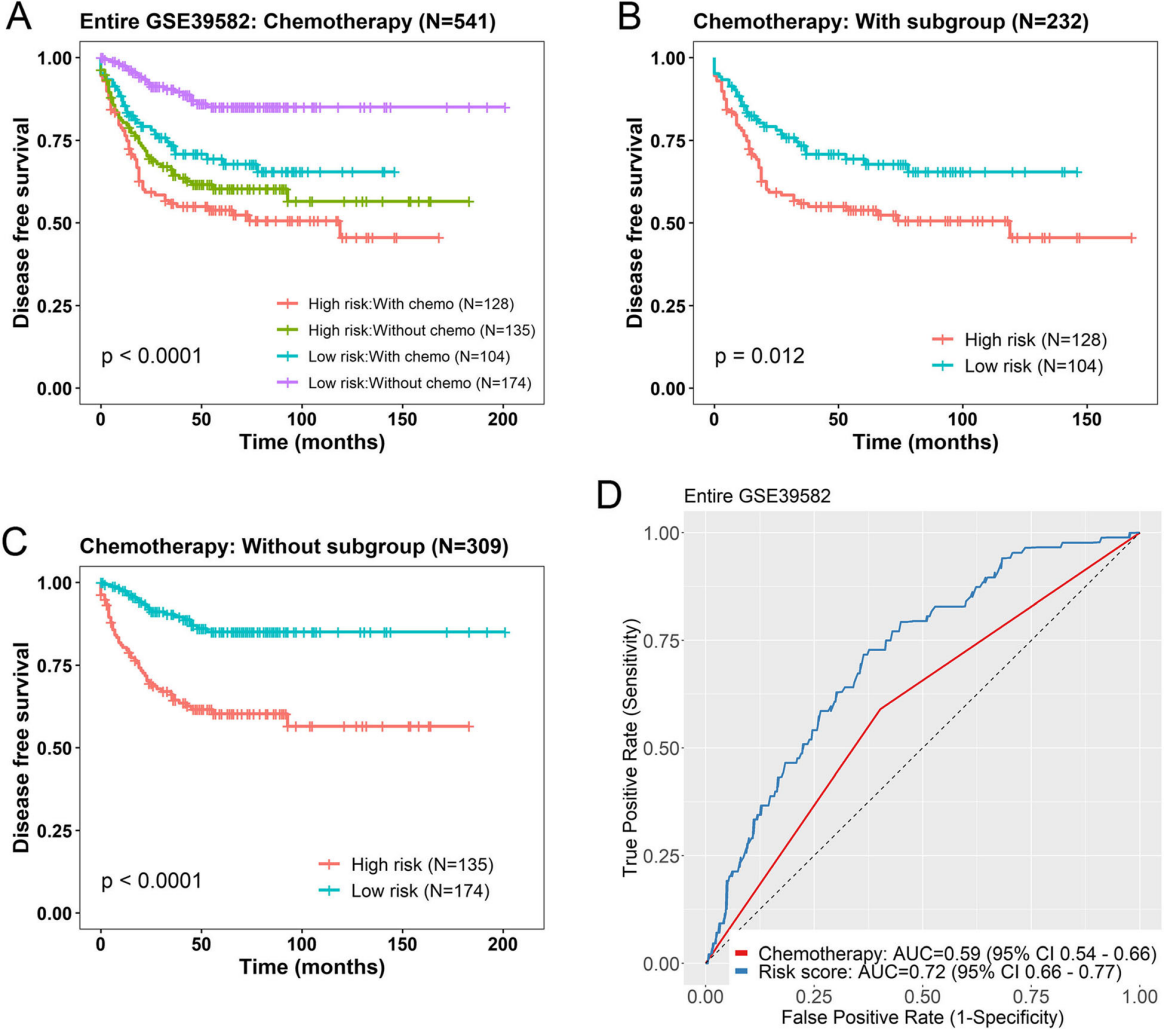
Kaplan-Meier estimates of the disease free survival (DFS) of patients using the seven-lncRNA signature, stratified by adjuvant chemotherapy. Entire GSE39582 dataset were first stratified by chemotherapy (with or without) and Kaplan-Meier plots were then used to visualize the survival probabilities for patients within each stratum. **a** Kaplan-Meier curves for the entire GSE39582 dataset patients (*N* = 541); **b** Kaplan-Meier curves for patients with adjuvant chemotherapy (*N* = 232); **c** Kaplan-Meier curves for patients without adjuvant chemotherapy (*N* = 309). The tick marks on the curves represent the censored events. The differences between the two curves were determined by the two-sided log-rank test. **d** The ROC curves of seven-lncRNA risk score (blue) and chemotherapy (red) were shown in entire GSE39582

Since adjuvant chemotherapy was significant in univariable Cox analysis (Additional file 2: Table S1), we then conducted multivariable Cox regression model on those 541 patients. Using risk score, adjuvant chemotherapy, age and gender as covariates, we found that the seven-lncRNA risk score (*P* < 0.001) and chemotherapy (*P* = 0.013) were both independent prognostic factors (Table 3). In addition, data stratification was performed, which stratified these patients into with chemotherapy or without chemotherapy subgroups. This analysis indicated that within each stratum, the seven-gene risk score could further identify patients with different prognoses (Fig. 5). For patients with chemotherapy (*N* = 232), the risk score could subdivide them into those likely to have longer DFS and those likely to have shorter DFS (log-rank test *P* = 0.012) (Fig. 5b). Patients without chemotherapy (*N* = 309) acted in a similar fashion (log-rank test *P* < 0.001) (Fig. 5c). The ROC analysis was performed and curves were visualized for the two factors. The AUC for the seven-lncRNA signature and chemotherapy on DFS was 0.72 and 0.59, respectively, indicating a favorable prognostic value in predict patients’ survival (Fig. 5d).

**Table 3:** Multivariable Cox regression analysis of the seven-lncRNA risk score and other variables in GSE39582 data set

Variables	HR	95% CI of HR	*P* value^a^
Seven-lncRNA risk score (*N* = 541)	2.14	1.783–2.568	< 0.001
Age	1.008	0.995–1.021	0.223
Gender	1.391	1.013–1.91	0.041
Adjuvant chemotherapy	1.522	1.091–2.124	0.013
Seven-lncRNA risk score (*N* = 536)	2.345	1.967–2.797	< 0.001
Age	0.996	0.984–1.008	0.469
Gender	1.35	0.99–1.842	0.058
KRAS mutation	0.691	0.509–0.938	0.018
Seven-lncRNA risk score (*N* = 511)	2.195	1.853–2.6	< 0.001
Age	0.999	0.987–1.012	0.911
Gender	1.184	0.869–1.613	0.285
MMR status	2.617	1.378–4.97	0.003

Abbreviations: *HR* hazard ratio, *CI* confidence interval, *MMR* mismatch repair

In Cox regression analyses, risk score was evaluated as continuous variables, all other variables were evaluated as category variables

^a^*P* < 0.05 was considered statistically significant in all analyses

### The prognostic values of seven-lncRNA signature is independent of KRAS mutation

**Fig. 6:**
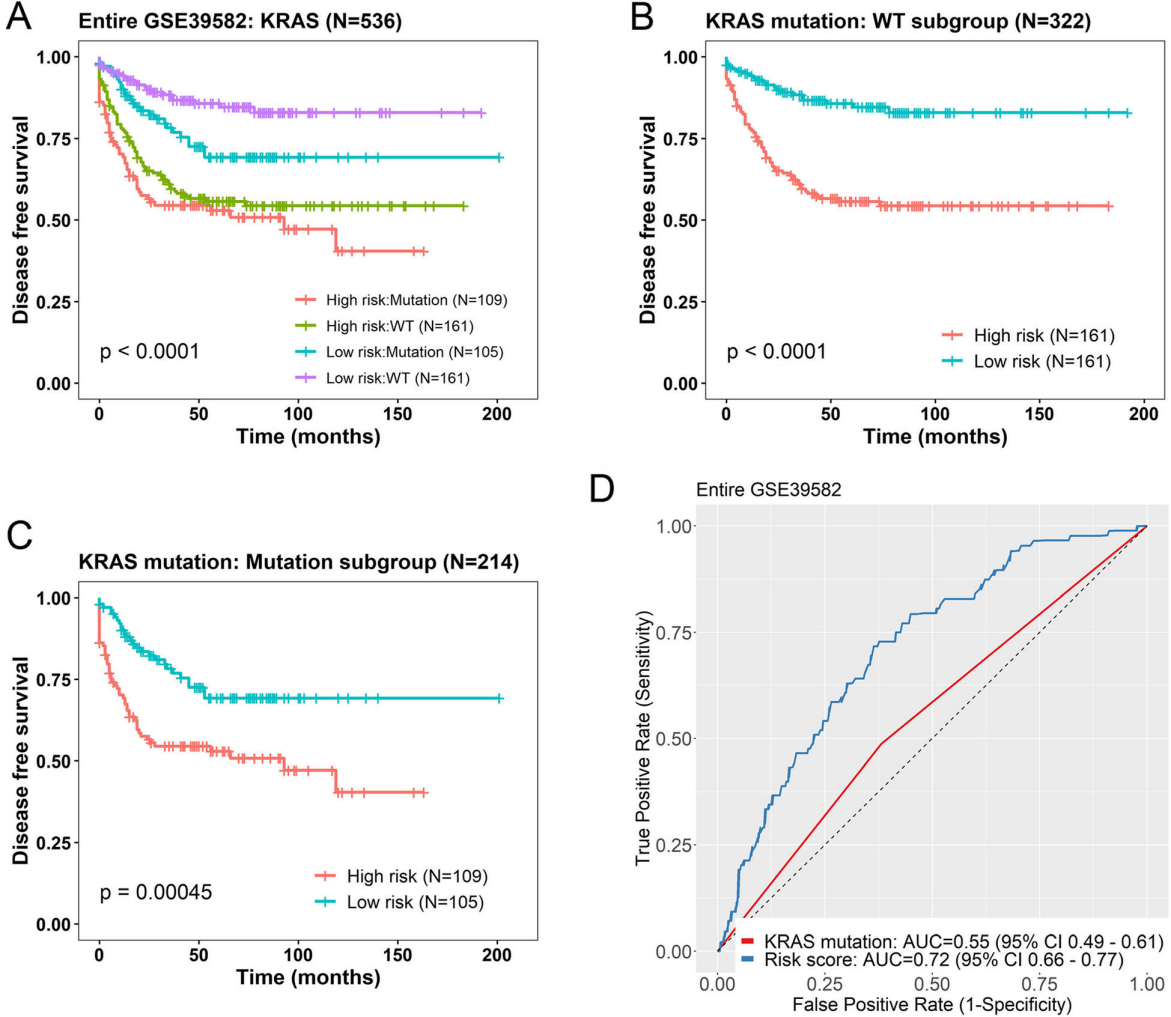
Kaplan-Meier estimates of the disease free survival (DFS) of patients using the seven-lncRNA signature, stratified by KRAS mutation status. Entire GSE39582 dataset were first stratified by KRAS mutation status (WT or Mutation) and Kaplan-Meier plots were then used to visualize the survival probabilities for patients within each stratum. **a** Kaplan-Meier curves for the entire GSE39582 dataset patients (*N* = 536); **b** Kaplan-Meier curves for patients with wide-type KRAS gene (WT, *N* = 322); **c** Kaplan-Meier curves for patients with mutated KRAS gene (Mutation, *N* = 214). The tick marks on the curves represent the censored events. The differences between the two curves were determined by the two-sided log-rank test. **d** The ROC curves of seven-lncRNA risk score (blue) and KRAS mutation (red) were shown in entire GSE39582

KRAS mutation occurs in 30 to 50% of colorectal cancers (CRCs) and has been suggested to be associated with proliferation and decreased apoptosis [31]. Thus, we tested whether the predictive power of the seven-lncRNA signature was independent of KRAS mutation status in GSE39582 (*N* = 536). In the multivariable Cox regression analysis, we found that the seven-lncRNA risk score (*P* < 0.0001) and KRAS mutation status (*P* = 0.018) were both independent prognostic factors (Table 3). In the stratification analysis, the seven-gene risk score could further identify patients with different prognoses (Fig. 6). For patients with WT KRAS (*N* = 322), the risk score could subdivide them into those likely to have longer DFS and those likely to have shorter DFS (log-rank test P < 0.0001) (Fig. 6b). Patients with KRAS mutation (*N* = 214) acted in a similar fashion when analyzed with this risk score (log-rank test *P* = 0.00045) (Fig. 6c). The ROC AUC for the seven-lncRNA signature was 0.72, which is much higher than that of KRAS mutation (0.55), indicating a better predictive ability (Fig. 6d).

We further tested the whether the predictive power of the seven-lncRNA signature was independent of MMR status in GSE39582 (*N* = 511). In the multivariable Cox regression analysis, the seven-lncRNA risk score (*P* < 0.0001) and MMR status (*P* = 0.003) were both independent prognostic factors (Table 3). In the stratification analysis, however, the risk score could identify subgroup patients with proficiency MMR but fail to divide deficiency MMR patients significantly (*P* = 0.092, Additional file 1: Figure S5). This may be because the sample size is too small (72 patients) to draw any firm conclusions. Concerning the chemotherapy, KRAS mutation and MMT status together, we further performed multivariable Cox analysis with the three factors (Additional file 2: Table S2). This analysis verified seven-lncRNA signature as an independent factor when putting KRAS mutation, chemotherapy and MMR status together (*P* < 0.001).

Although other clinical variables, such as TP53 mutation, were not significant in univariable Cox regression analysis (*P* > 0.05, Additional file 2: Table S1), we still performed multivariable Cox analysis to identify their possible associations with seven-lncRNA signature. As shown in Additional file 2: Table S3, the results demonstrated that the seven-lncRNA signature was independent of these clinical variables. Furthermore, we examined all clinical variables in one analysis of multivariable Cox regression and proved that the seven-lncRNA signature was an independent influencing factor of all the variables (Additional file 2: Table S4).

In addition, we further performed ROC analysis to compare the discrimination power between seven-lncRNA signature and all other available clinical features in GSE39582 data set. As shown in Additional file 1: Figure S6, The AUC for the seven-lncRNA signature was comparatively higher than all other factors. These above results indicated that the seven-lncRNA could be used as an effective prognostic signature for CRC patients.

### Identification of seven-lncRNA signature altered pathways

**Fig. 7:**
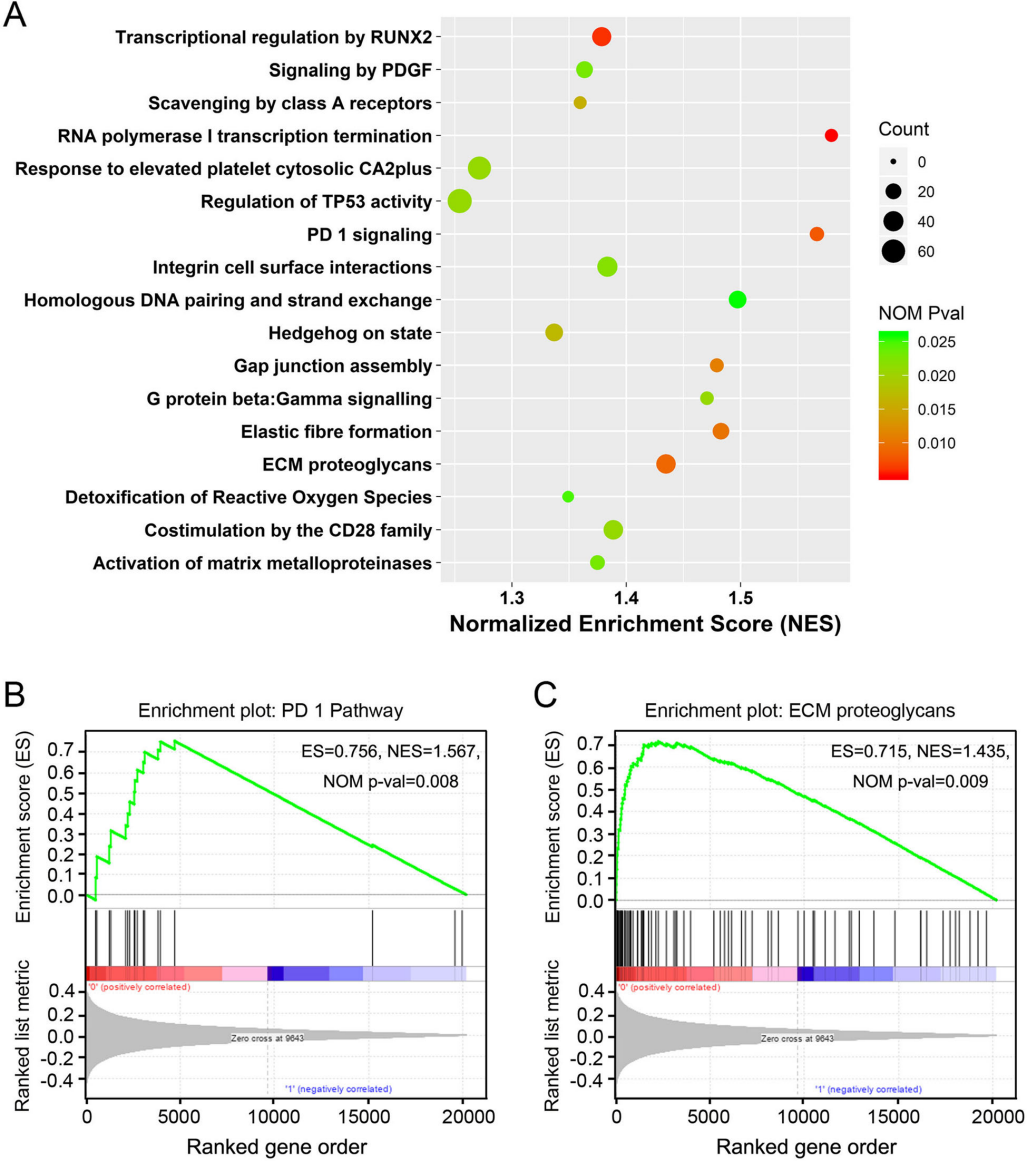
Gene set enrichment analysis identified altered signaling pathways related to risk score. **a** The scatter plot shown pathways enriched in high risk score group patients of GSE39582. Of those, the enrichment plots of “PD 1 pathway” **b** and “ECM proteoglycans” **c** were shown. ES, enrichment score; NES, normalized ES; NOM Pval, normalized *p*-value

To identify potentially altered signaling pathways, we performed GSEA using the seven-lncRNA signature based risk score classification. Samples from GSE39582 (*N* = 557) were classified into high risk (*N* = 278) or low risk group (*N* = 279) using the median risk score. According to the results, we found that some Reactome pathways were significantly enriched (normalized *P* value < 0.05, Fig. 7a, Additional file 3: Table S5). Of these, several pathways were noticed for their roles in tumorigenesis and tumor progression, including “Integrin cell surface interactions” and “Activation of matrix metalloproteinases”. The enrichment plots of “PD 1 pathway” and “ECM proteoglycans” were shown as examples (Fig. 7b, c). These results suggested that the seven lncRNA based risk score may reflect the status of these signaling pathways.

Additionally, it should be useful to investigate the potential molecular networks that seven lncRNAs were commonly associated with. We searched interacted proteins and miRNAs in ENCORI database (starBase v3.0) [31] and identified networks of the seven lncRNAs may interacted with (Additional file 4: Table S6). As shown in Additional file 1: Figure S7, totally 55 proteins were found to be associated with six lncRNAs and 87 microRNAs were associated with three lncRNAs. Although these findings needed to be further verified, they implicated possible networks for future biological studies of these lncRNAs.

## Discussion

LncRNAs were proved to be indispensable in comprehensive biological processes through different mechanisms. In recent years increasing evidence has demonstrated that lncRNAs may play important roles in tumorigenesis and tumor progression [5, 6]. More recently, lncRNAs have been implicated in the pathogenesis and prognosis of CRC [25, 26]. The investigating of prognostic potential of lncRNAs in CRC is of great value. In the present study, we identified a potential seven-lncRNA signature that was significantly associated with the DFS of CRC patients. Two GEO datasets were employed in this study. After a comprehensive analysis, a seven-lncRNA signature was identified for predicting prognosis of CRC patients. Furthermore, Cox regression, stratification and ROC analysis suggested that the seven-lncRNA signature had a high predictive accuracy in our analyses.

For these seven lncRNAs, six of them (CTD-2354A18.1, NR2F1-AS1, AC073283.1, MIR31HG, AL132709.8, RP11-834C11.4) acted as risk factors for CRC, while AC069278.4 was protective factor (Table 1). We searched the literature to characterize these lncRNAs, finding three of them have been reported to correlated with cancer. CTD-2354A18.1 has been reported to be differentially expressed and may play a key role in the pathogenesis of gastric cancer [33]. In addition, it was regarded to be related to overall survival in CRC patients [34]. NR2F1-AS1 was shown to be upregulated in multiple cancer, including hepatocellular carcinoma, endometrial cancer, thyroid cancer and esophageal squamous cell carcinoma [35–38]. These different studies have revealed that NR2F1-AS1 can promote cancer progression via interacting with several miRNA and through different signaling pathways, including Hedgehog signaling pathway and PI3K/AKT pathway.

Another candidate, MIR31HG gene was thoroughly investigated according to the literature. Montes et al. suggested that MIR31HG could regulate INK4A expression to modulate senescence [39]. And Jin et al. indicated that Inhibition of MIR31HG promotes osteogenesis of human adipose-derived stem cells [40]. MIR31HG was also reported to be involved in the progression of multiple cancer, including bladder cancer, pancreatic ductal adenocarcinoma, esophageal squamous cell carcinoma, lung cancer, and et al. [41–43]. In addition, MIR31HG was strongly correlated with miR-31 expression, associating with poor outcome in CRC [43]. Thus, we infer that MIR31HG may act as an oncogene in CRC tumorigenesis and further investigations are needed as well. For the rest four lncRNAs, they were either poorly investigated or have not been reported at all. For example, although there were no experimental evidences about its function or mechanism in cancer, AL132709.8 was identified as a potential biomarker associated with recurrence of ovarian cancer [44]. All the above reports provided us the opportunity to better understand the roles that might be played by these lncRNAs.

The seven-lncRNA signature is an independent prognostic factors in CRC. Pathological staging is widely used to classify patients for adjuvant chemotherapy in clinical [15]. Despite of this, appreciable efforts have been made in the past decades to discover the molecular biomarkers that may serve as a determinant to subclass CRC patients [45, 46]. These studies have determined a series of biomarkers that are thought to be associated with the prognosis of CRC, including MMR status, KRAS mutation, BRAF mutation and et al. [45]. Among these biomarkers, microsatellite instability, caused by dysfunction of the DNA repair system, is the only marker that was found to be a significant prognostic factor in early CRC [47]. Therefore, it will be highly desirable to evaluate whether the prognostic value of the seven-lncRNA signature is independent of these well-recognized factors. Here, we found the seven-lncRNA signature was independent of each TNM stage and had a similar ROC AUC value as TNM stage (Table 2). Stratification analysis demonstrated this signature was significantly associated with DFS in patients with stage IV. We assessed the association between all available clinical variables and subsequent multivariate Cox regression and stratification analysis indicated that the prognostic value of our seven-lncRNA signature was independent of these factors.

We also performed GSEA to identify the potential biological pathways altered between the high- and low-risk patients. Enriched pathways were noticed for their roles in tumorigenesis and tumor progression. The above findings suggested a possible function role played by these seven lncRNAs. To reveal the potential molecular mechanism of these lncRNAs, we established their interaction networks of proteins and miRNAs. However, only six and three of them were identified in the ENCORI database to be associated with proteins and miRNAs, respectively. Although the roles of these lncRNAs in CRC or other disease are currently unclear, our findings implicated that their molecular mechanism deserve further investigation.

However, there are several limitations in our study. Firstly, lncRNAs profiled in this study were re-annotated from Affymetrix Human Genome U133 platform, which probably only represents part of the lncRNA populations. So the lncRNA signature identified here may not represent the most significant one in CRC. Secondly, we have no experimental data and shortage of information on the mechanisms behind the prognostic values of these seven lncRNAs. Although the function of some lncRNAs has been reported, more efforts need to be made to further understand their role in CRC. Finally, we suggested that the lncRNA signature is independent of other features, such as KRAS mutation and adjuvant chemotherapy. Unfortunately, this conclusion can only be tested in GSE39582 data set, because there is no such clinical information in GSE17528 cohort.

## Conclusions

In summary, by employing large independent patient cohorts, we identified a seven-lncRNA signature to predict the DFS of CRC patients. The seven-lncRNA signature showed great potential of prognostic prediction and independent of several well acknowledged factors. Although these findings needed to be further investigated, they illustrated a promising perspective in the development of prognostic biomarkers and showed useful implications for future biological studies.

## Supplementary information

**Additional file 1 Figure S1.** Schematic of the study. **Figure S2.** LncRNA risk score performance in the GSE39582 validation dataset. The distribution of signature risk score, patients’ survival status and sevenlncRNA expression were analyzed in the GSE39582 validation patients (*N* = 278). (A) The distribution of lncRNA signature risk score distribution; (B) The survival status and time of corresponding patient; (C) The heatmap of the lncRNA expression value. Rows represent lncRNAs and columns for patients. The black line means the median risk score cutoff dividing patients into low-risk and high-risk groups. **Figure S3.** LncRNA risk score performance in the entire GSE39582 dataset. The distribution of signature risk score, patients’ survival status and seven-lncRNA expression were analyzed in the entire GSE39582 patients (*N* = 557). (A) The distribution of lncRNA signature risk score distribution; (B) The survival status and time of corresponding patient; (C) The heatmap of the lncRNA expression value. Rows represent lncRNAs and columns for patients. The black line means the median risk score cutoff dividing patients into low-risk and high-risk groups. **Figure S4.** LncRNA risk score performance in the GSE17538 validation dataset. The distribution of signature risk score, patients’ survival status and sevenlncRNA expression were analyzed in the GSE17538 validation patients(*N* = 200). (A) The distribution of lncRNA signature risk score distribution; (B) The survival status and time of corresponding patient; (C) The heatmap of the lncRNA expression value. Rows represent lncRNAs and columns for patients. The black line means the median risk score cutoff dividing patients into low-risk and high-risk groups. **Figure S5.** Kaplan-Meier estimates of the disease free survival (DFS) of patients using the seven-lncRNA signature, stratified by MMR status. Entire GSE39582 dataset were first stratified by MMR status (dMMR or pMMR) and Kaplan-Meier plots were then used to visualize the survival probabilities for patients within each stratum. (A) Kaplan-Meier curves for the entire GSE39582 dataset patients (*N* = 511); (B) Kaplan-Meier curves for patients with dMMR (*N* = 72); (C) Kaplan-Meier curves for patients with pMMR (*N* = 439). The tick marks on the curves represent the censored events. The differences between the two curves were determined by the two-sided log-rank test. dMMR, deficient mismatch repair; pMMR, proficient mismatch repair. (D) The ROC curves of sevenlncRNA risk score (blue) and MMR status (red) were shown in entire GSE39582. **Figure S6.** Receiver operating characteristic (ROC) analysis of Seven-lncRNA risk score and other available clinical features in entire GSE39582 data set. Patients with known information about CIMP status (*N* = 487), BRAF mutation (*N* = 503), CIN status (*N* = 455), CIT subtype (*N* = 537), TP53 mutation (*N* = 344), Tumorlocation (*N* = 557) were evaluated. CIMP, CpG island methylator phenotype; CIN, chromosomal instability; CIT subtype, Cartes d’Identite des Tumeurs molecular subtype. **Figure S7.** LncRNAs interaction networks. (A) The network represents lncRNAs (Yellow diamond) and associated proteins (Purple circle), in which 6 lncRNAs and 55 proteins derived from CLIP-seq data from ENCORI were visualized. (B) The network represents lncRNAs (Yellow diamond) and interacted miRNA (Purple circle), in which 3 lncRNAs and 87 miRNAs derived from ENCORI were shown.

**Additional file 2 Table S1** Univariable Cox regression analysis of the seven-lncRNA risk score and other available variables in GSE39582 data set. **Table S2** Multivariable Cox regression analysis of the seven-lncRNA risk score and five other variables in GSE39582 data set (*N* = 473). **Table S3** Multivariable Cox regression analysis of the seven-lncRNA risk score and other variables in GSE39582 data set. **Table S4** Multivariable Cox regression analysis of the seven-lncRNA risk score and all eleven available clinical variables in GSE39582 data set (*N* = 249).

**Additional file 3 Table S5.** Gene set enrichment analysis (GSEA) results of GSE39582 cohort.

**Additional file 4 Table S6A.** LncRNAs associated protein identified in ENCORI database. Table S6B. LncRNAs associated miRNAs identified in ENCORI database.

## Data Availability

The data sets used and/or analyzed during the current study are publicly available data from Gene Expression Omnibus (GEO) databases (GSE39582, GSE17538). The figures and materials supporting the conclusions of this article are included within the article and its additional files).

## Abbreviations

AUC: Area under the curve
BRB-Array: Biometric Research Branch-Array
CI: Confidence interval
CIMP: CpG island methylator phenotype
CIN: Chromosomal instability
CRC: Colorectal cancer
DFS: Disease free survival
GCRMA: Guanine Cytosine Robust Multi-Array Average method
GEO: Gene expression Ominus
GSEA: Gene set enrichment analysis
HR: Hazard ratio
lncRNAs: Long non-coding RNAs
miRNA: microRNA
MMR: Mismatch repair
NA: Not Available
ROC: Receiver operating characteristic
