# Supplementary material for: LncRNA profile study reveals a seven-lncRNA signature predicts the prognosis of patients with colorectal cancer
Source: Biomark Res. 2020 Feb 28;8:8. doi: 10.1186/s40364-020-00187-3 (PMC7047379; doi:10.1186/s40364-020-00187-3)
Supplement: Supplementary file 1 — Additional file 1 Figure S1. Schematic of the study. Figure S2. LncRNA risk score performance in the GSE39582 validation dataset. The distribution of signature risk score, patients’ survival status and sevenlncRNA expression were analyzed in the GSE39582 validation patients (N = 278). (A) The distribution of lncRNA signature risk score distribution; (B) The survival status and time of corresponding patient; (C) The heatmap of the lncRNA expression value. Rows represent lncRNAs and columns for patients. The black line means the median risk score cutoff dividing patients into low-risk and high-risk groups. Figure S3. LncRNA risk score performance in the entire GSE39582 dataset. The distribution of signature risk score, patients’ survival status and seven-lncRNA expression were analyzed in the entire GSE39582 patients (N = 557). (A) The distribution of lncRNA signature risk score distribution; (B) The survival status and time of corresponding patient; (C) The heatmap of the lncRNA expression value. Rows represent lncRNAs and columns for patients. The black line means the median risk score cutoff dividing patients into low-risk and high-risk groups. Figure S4. LncRNA risk score performance in the GSE17538 validation dataset. The distribution of signature risk score, patients’ survival status and sevenlncRNA expression were analyzed in the GSE17538 validation patients(N = 200). (A) The distribution of lncRNA signature risk score distribution; (B) The survival status and time of corresponding patient; (C) The heatmap of the lncRNA expression value. Rows represent lncRNAs and columns for patients. The black line means the median risk score cutoff dividing patients into low-risk and high-risk groups. Figure S5. Kaplan-Meier estimates of the disease free survival (DFS) of patients using the seven-lncRNA signature, stratified by MMR status. Entire GSE39582 dataset were first stratified by MMR status (dMMR or pMMR) and Kaplan-Meier plots were then used to visualize the surviva [file 40364_2020_187_MOESM1_ESM.pdf]

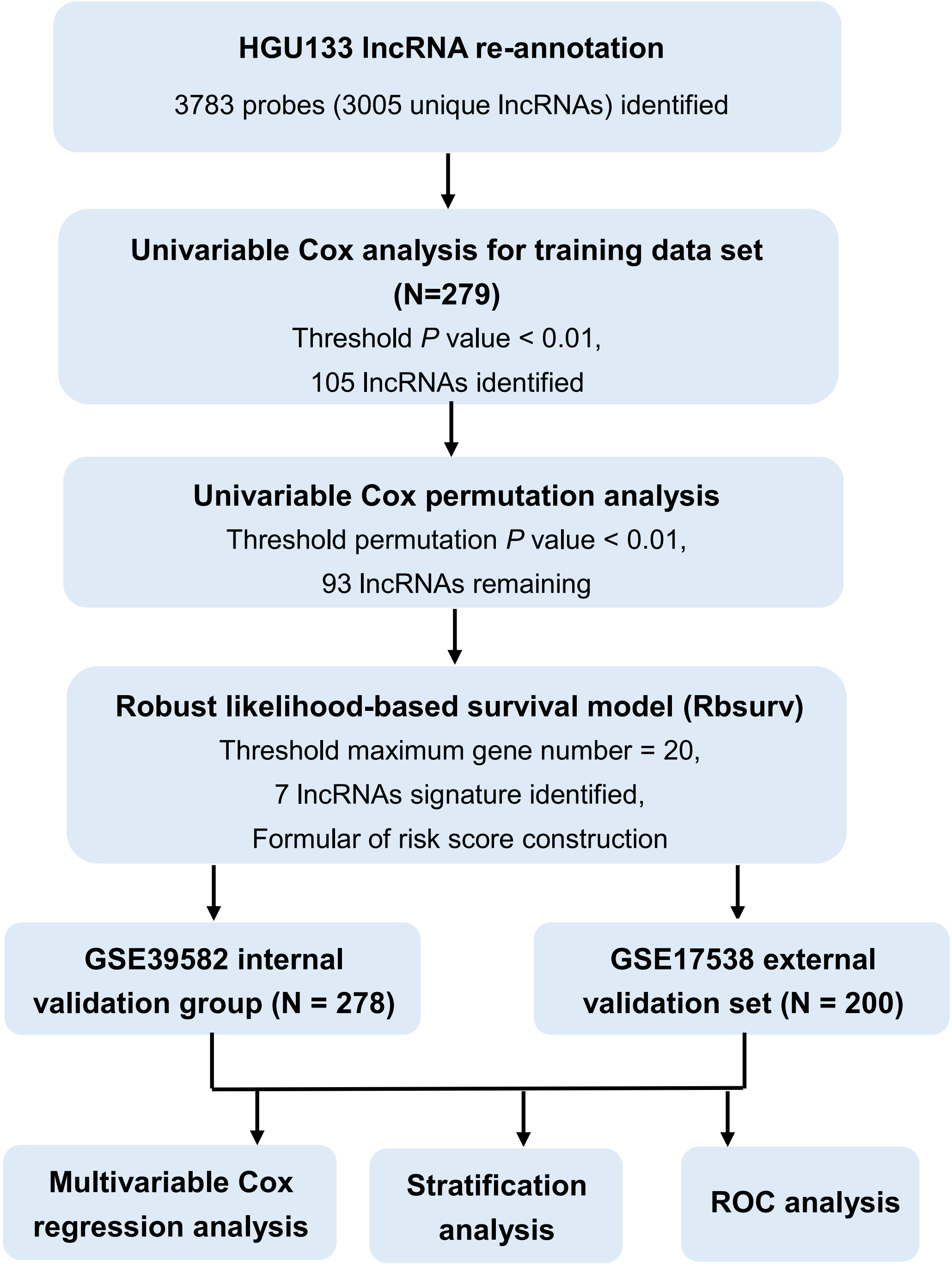

Fig. S1 Schematic of the study.

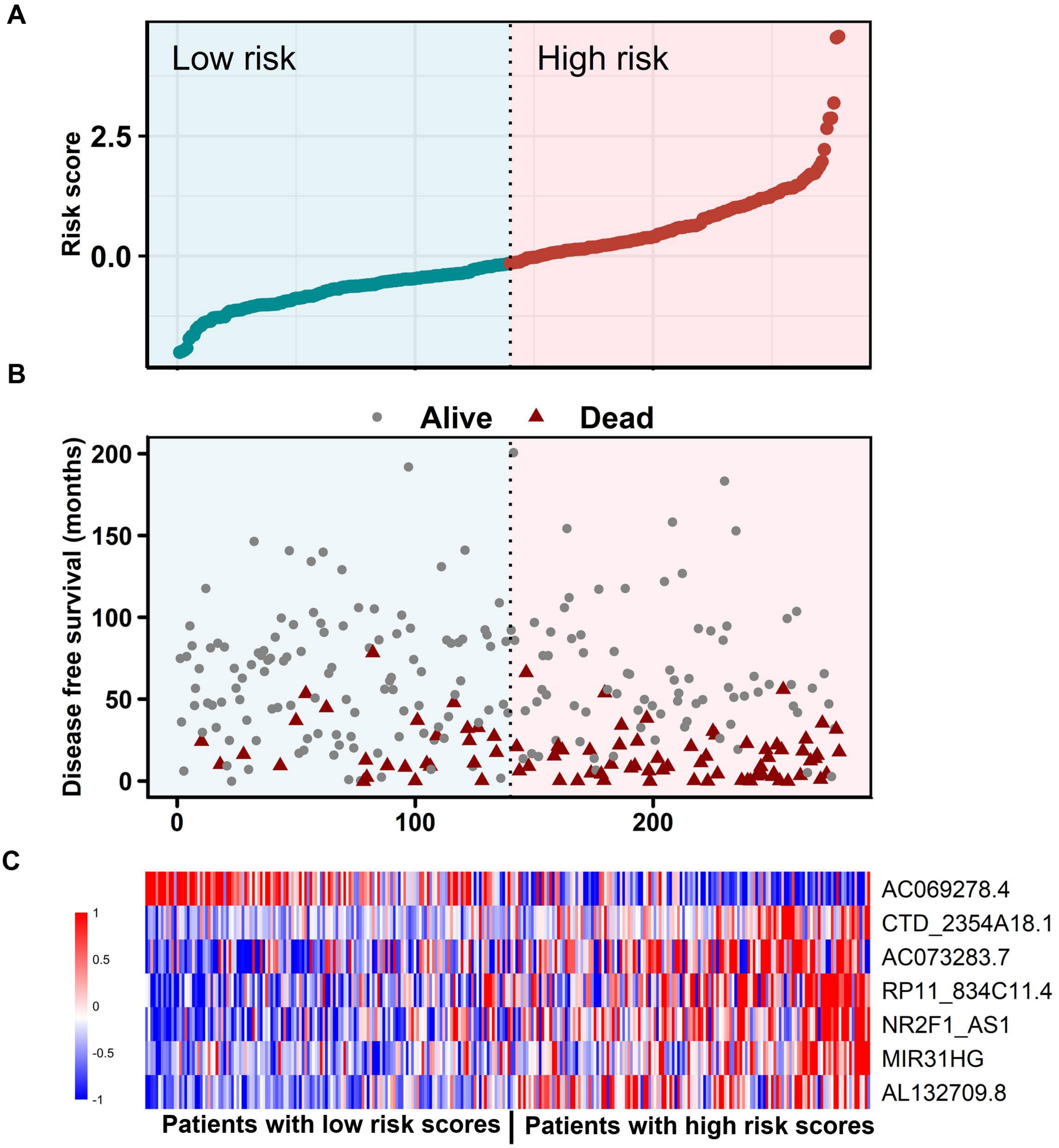

Fig. S2. LncRNA risk score performance in the GSE39582 validation dataset.

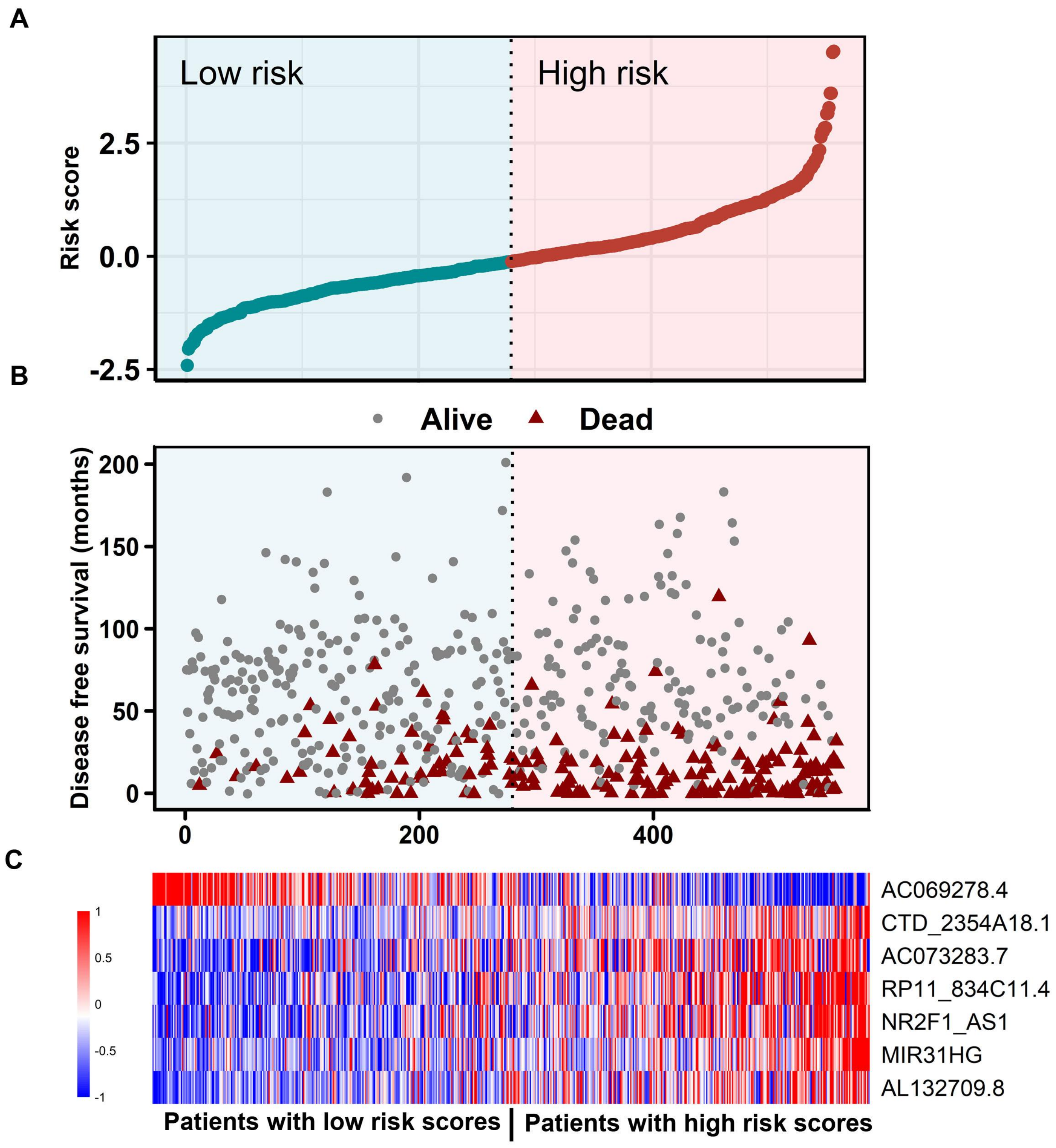

Fig. S3. LncRNA risk score performance in the entire GSE39582 dataset.

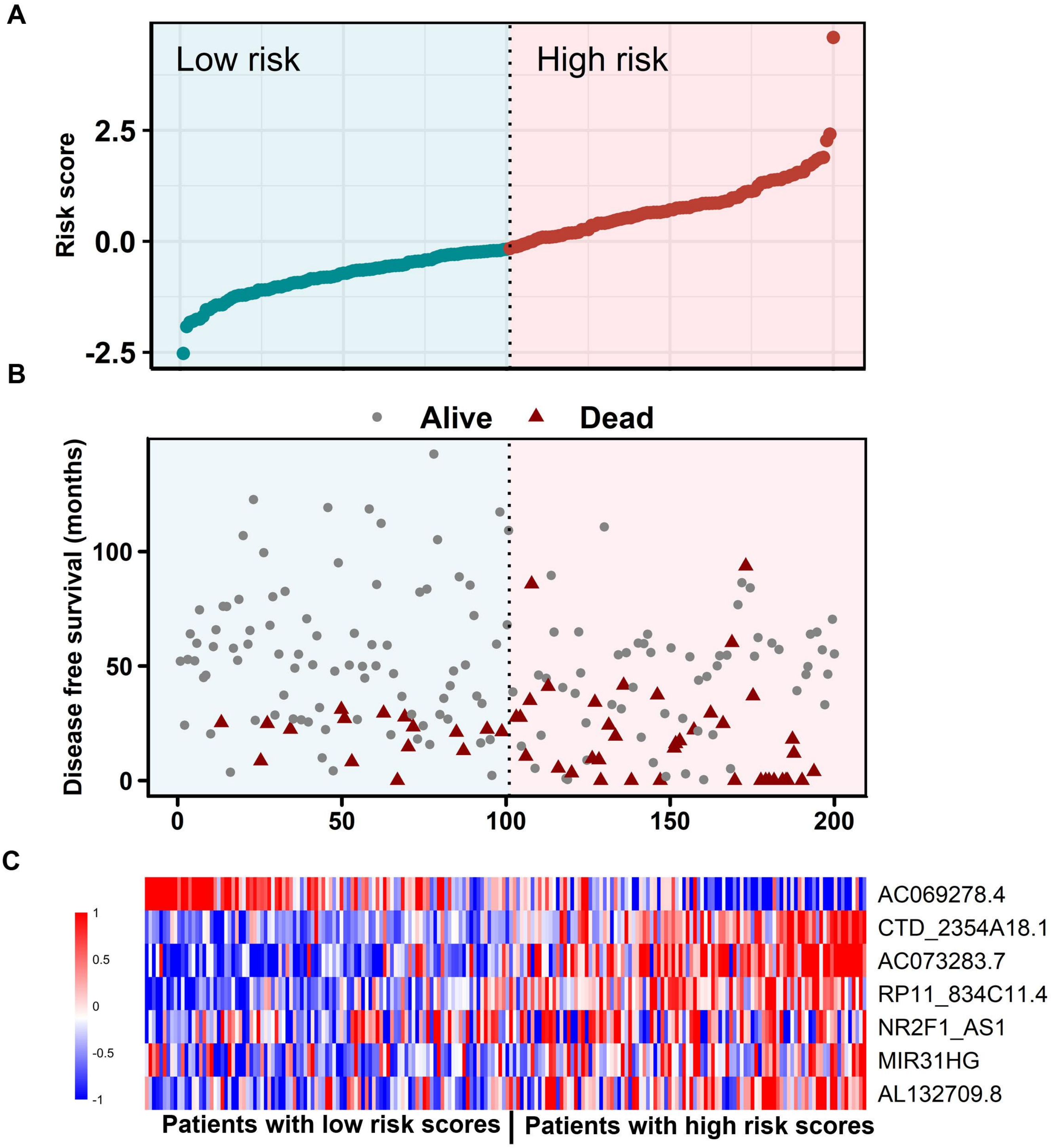

Fig. S4. LncRNA risk score performance in the GSE17538 validation dataset.

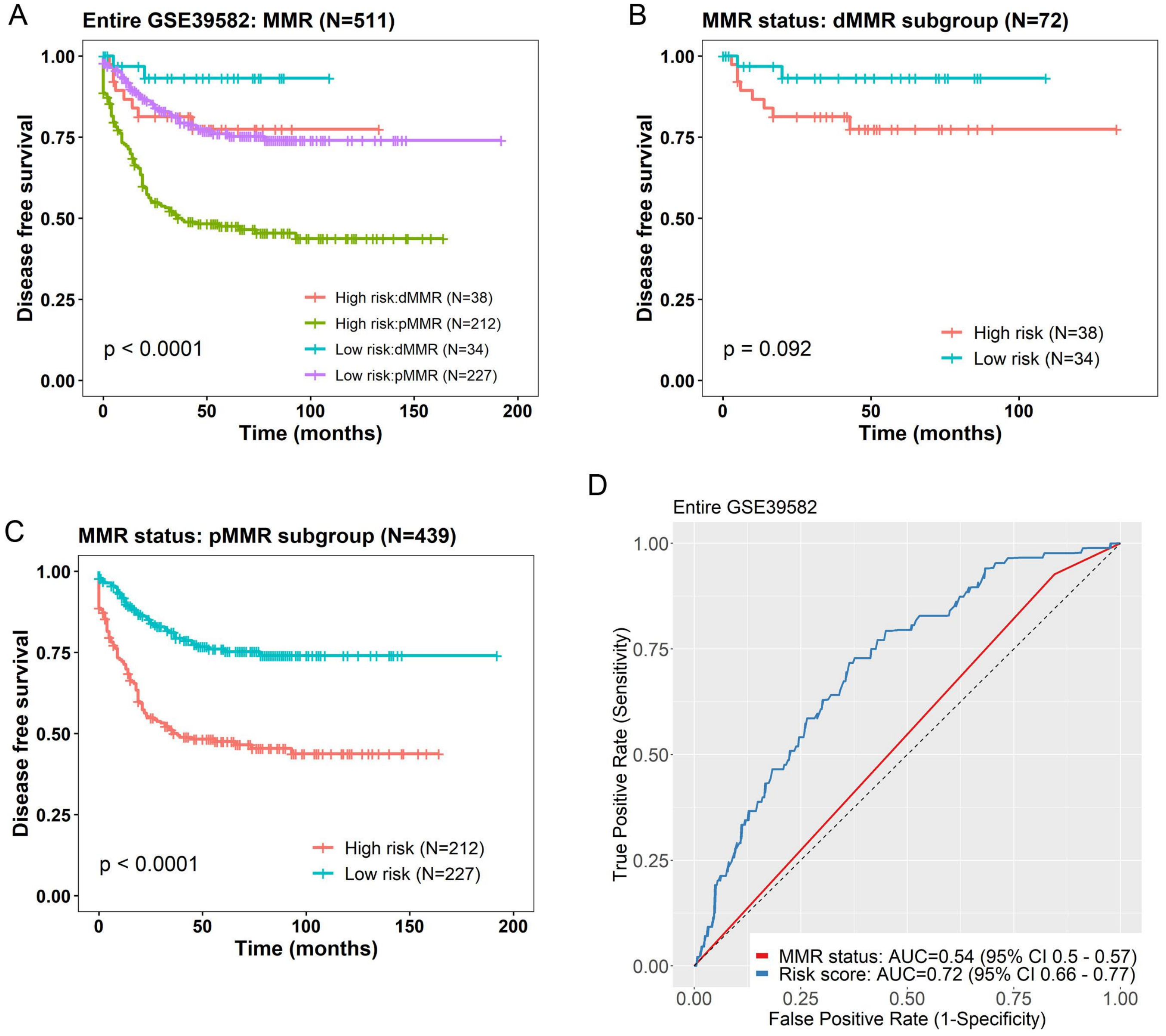

Fig. S5. Kaplan-Meier estimates of the disease free survival (DFS) of patients using the seven-lncRNA signature, stratified by MMR status.

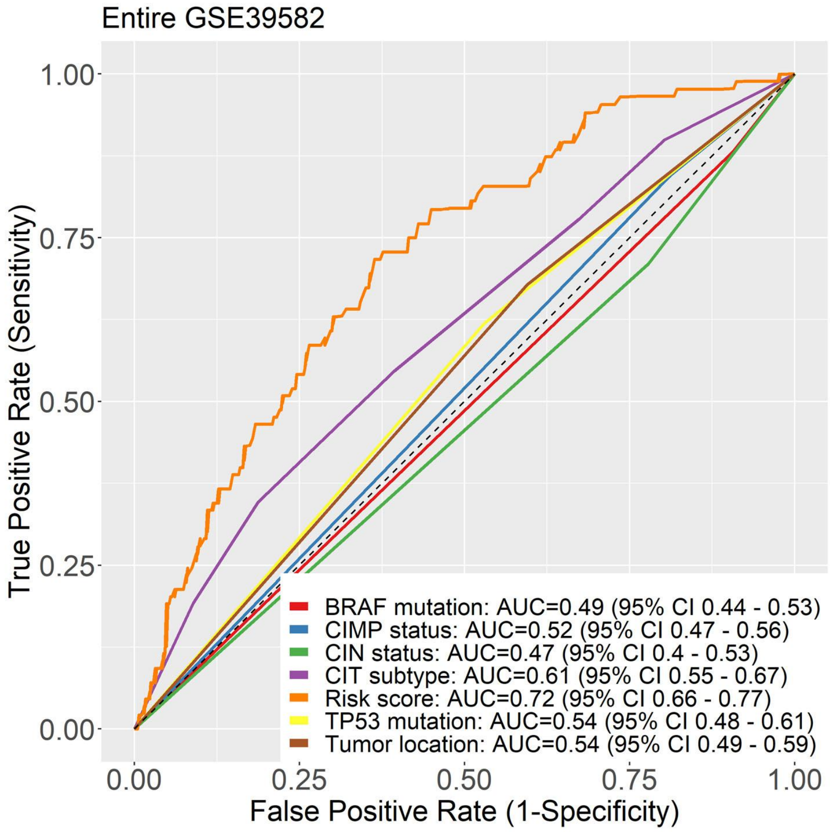

Fig. S6. Receiver operating characteristic (ROC) analysis of Seven-lncRNA risk score and other available clinical features in entire GSE39582 data set.

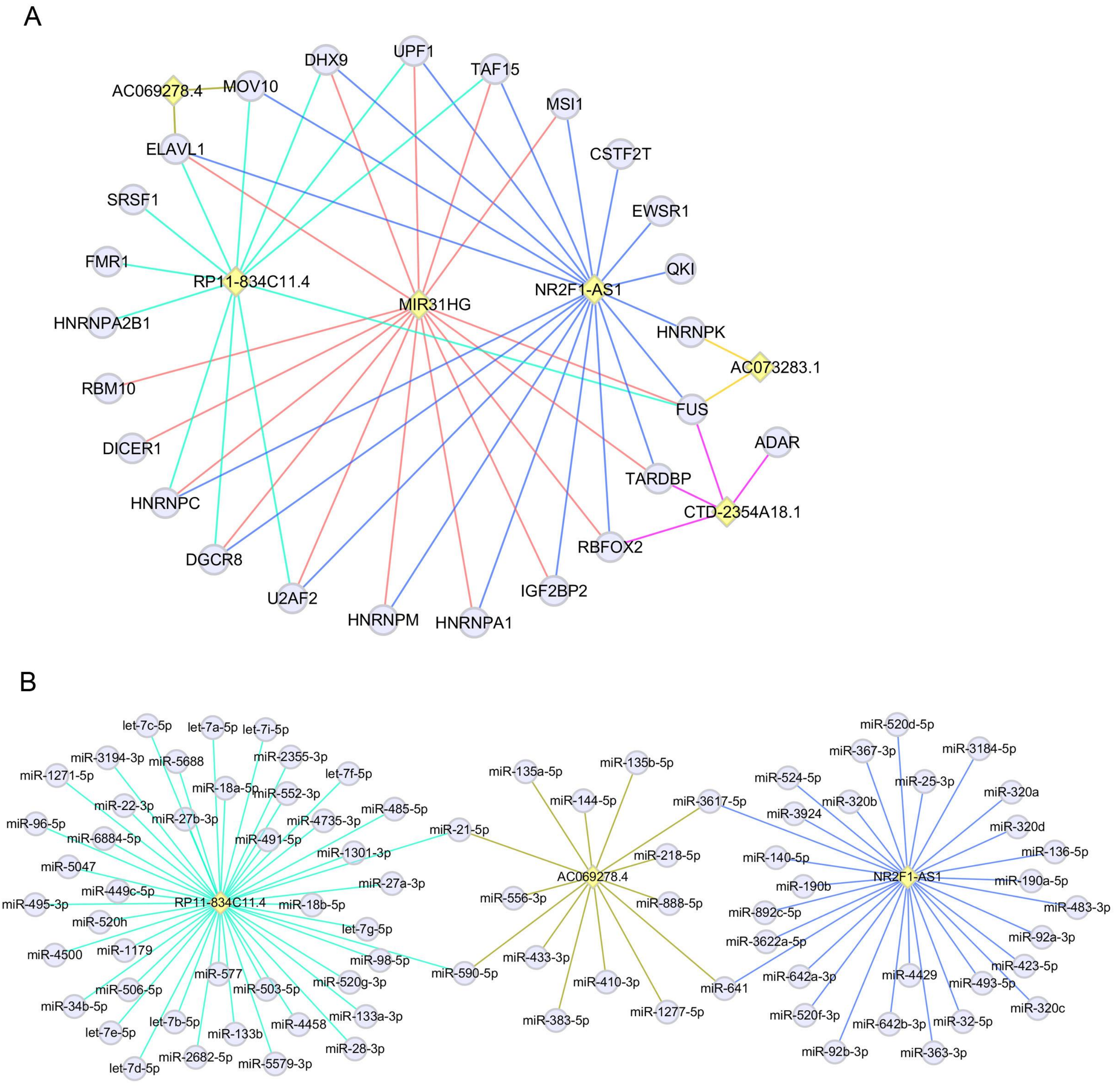

Fig. S7. LncRNA interaction networks. (A) Six lncRNAs (Yellow diamond) and asscociated 55 proteins (Purple circle). (B) Three lncRNAs (Yellow diamond) and 87 interacted miRNA (Purple circle).

Fig. S1. Schematic of the study.

Fig. S2. LncRNA risk score performance in the GSE39582 validation dataset. The distribution of signature risk score, patients' survival status and seven-lncRNA expression were analyzed in the GSE39582 validation patients (N = 278). (A) The distribution of lncRNA signature risk score distribution; (B) The survival status and time of corresponding patient; (C) The heatmap of the lncRNA expression value. Rows represent lncRNAs and columns for patients. The black line means the median risk score cutoff dividing patients into low-risk and high-risk groups.

Fig. S3. LncRNA risk score performance in the entire GSE39582 dataset. The distribution of signature risk score, patients' survival status and seven-lncRNA expression were analyzed in the entire GSE39582 patients (N = 557). (A) The distribution of lncRNA signature risk score distribution; (B) The survival status and time of corresponding patient; (C) The heatmap of the lncRNA expression value. Rows represent lncRNAs and columns for patients. The black line means the median risk score cutoff dividing patients into low-risk and high-risk groups.

Fig. S4. LncRNA risk score performance in the GSE17538 validation dataset. The distribution of signature risk score, patients' survival status and seven-lncRNA expression were analyzed in the GSE17538 validation patients

(N = 200). (A) The distribution of lncRNA signature risk score distribution; (B) The survival status and time of corresponding patient; (C) The heatmap of the lncRNA expression value. Rows represent lncRNAs and columns for patients. The black line means the median risk score cutoff dividing patients into low-risk and high-risk groups.

Fig. S5. Kaplan-Meier estimates of the disease free survival (DFS) of patients using the seven-lncRNA signature, stratified by MMR status. Entire GSE39582 dataset were first stratified by MMR status (dMMR or pMMR) and Kaplan-Meier plots were then used to visualize the survival probabilities for patients within each stratum. (A) Kaplan-Meier curves for the entire GSE39582 dataset patients (N = 511); (B) Kaplan-Meier curves for patients with dMMR (N = 72); (C) Kaplan-Meier curves for patients with pMMR (N = 439). The tick marks on the curves represent the censored events. The differences between the two curves were determined by the two-sided log-rank test. dMMR, deficient mismatch repair; pMMR, proficient mismatch repair. (D) The ROC curves of seven-lncRNA risk score (blue) and MMR status (red) were shown in entire GSE39582.

Fig. S6. Receiver operating characteristic (ROC) analysis of Seven-lncRNA risk score and other available clinical features in entire GSE39582 data set. Patients with known information about CIMP status (N = 487), BRAF mutation (N = 503), CIN status (N = 455), CIT subtype (N = 537), TP53 mutation (N = 344), Tumor

location (N = 557) were evaluated. CIMP, CpG island methylator phenotype; CIN, chromosomal instability; CIT subtype, Cartes d'Identite des Tumeurs molecular subtype.

Fig. S7. LncRNAs interaction networks. (A) The network represents lncRNAs (Yellow diamond) and associated proteins (Purple circle), in which 6 lncRNAs and 55 proteins derived from CLIP-seq data from ENCORI were visualized. (B) The network represents lncRNAs (Yellow diamond) and interacted miRNA (Purple circle), in which 3 lncRNAs and 87 miRNAs derived from ENCORI were shown.
