# Supplementary material for: LncRNA profile study reveals a seven-lncRNA signature predicts the prognosis of patients with colorectal cancer
Source: Biomark Res. 2020 Feb 28;8:8. doi: 10.1186/s40364-020-00187-3 (PMC7047379; doi:10.1186/s40364-020-00187-3)
Supplement: Supplementary file 2 — Additional file 2 Table S1 Univariable Cox regression analysis of the seven-lncRNA risk score and other available variables in GSE39582 data set. Table S2 Multivariable Cox regression analysis of the seven-lncRNA risk score and five other variables in GSE39582 data set (N = 473). Table S3 Multivariable Cox regression analysis of the seven-lncRNA risk score and other variables in GSE39582 data set. Table S4 Multivariable Cox regression analysis of the seven-lncRNA risk score and all eleven available clinical variables in GSE39582 data set (N = 249). [file 40364_2020_187_MOESM2_ESM.docx]

| **Table S1** Univariable Cox regression analysis of the seven-lncRNA risk score and other available variables in GSE39582 data set. | | | |
| --- | --- | --- | --- |
| **Variables** | **HR** | **95% CI of HR** | ***P* value^a^** |
| Seven-lncRNA risk score (*N* = 557) | 2.275 | 1.918-2.699 | <0.001 |
| Age (*N* = 557) | 0.999 | 0.988-1.01 | 0.882 |
| Gender (*N* = 557) | 1.267 | 0.938-1.712 | 0.123 |
| Stage (*N* = 557) | 2.687 | 2.172-3.324 | <0.001 |
| Chemotherapy (*N* = 541) | 1.816 | 1.332-2.478 | <0.001 |
| MMR status (*N* = 511) | 2.812 | 1.484-5.33 | 0.00153 |
| KRAS mutation (*N* = 536) | 0.705 | 0.521-0.954 | 0.0234 |
| Tumor location (*N* = 557) | 0.774 | 0.567-1.057 | 0.107 |
| TP53 mutation (*N* = 344) | 0.734 | 0.516-1.044 | 0.0854 |
| CIMP (*N* = 487) | 0.688 | 0.425-1.116 | 0.13 |
| CIN status (*N* = 455) | 1.126 | 0.72-1.761 | 0.603 |
| BRAF mutation (*N* = 503) | 1.073 | 0.607-1.895 | 0.809 |
| Abbreviations: HR, hazard ratio; CI, conﬁdence interval; CIMP, CpG island methylator phenotype; MMR, mismatch repair; CIN, chromosomal instability.  In Cox regression analyses, risk score and age was evaluated as continuous variables, all other variables were evaluated as category variables.  ^a^ *P* < 0.05 was considered statistically signiﬁcant in all analyses. | | | |

| **Table S2** Multivariable Cox regression analysis of the seven-lncRNA risk score and five other variables in GSE39582 data set (*N* = 473). | | | |
| --- | --- | --- | --- |
| **Variables** | **HR** | **95% CI of HR** | ***P* value^a^** |
| Seven-lncRNA risk score | 2.28 | 1.879-2.766 | <0.001 |
| Age | 0.999 | 0.985-1.013 | 0.871 |
| Gender | 1.261 | 0.898-1.771 | 0.181 |
| Adjuvant chemotherapy | 0.958 | 0.665-1.38 | 0.817 |
| KRAS mutation | 0.755 | 0.541-1.054 | 0.099 |
| MMR status | 2.507 | 1.26-4.988 | 0.009 |
| Abbreviations: HR, hazard ratio; CI, conﬁdence interval; MMR, mismatch repair.  In Cox regression analyses, risk score was evaluated as continuous variables, all other variables were evaluated as category variables.  ^a^ *P* < 0.05 was considered statistically signiﬁcant in all analyses. | | | |

| **Table S3** Multivariable Cox regression analysis of the seven-lncRNA risk score and other variables in GSE39582 data set. | | | |
| --- | --- | --- | --- |
| **Variables** | **HR** | **95% CI of HR** | ***P* value^a^** |
| Seven-lncRNA risk score (*N* = 344) | 2.466 | 1.988-3.059 | <0.001 |
| Age | 1.001 | 0.987-1.016 | 0.861 |
| Gender | 1.336 | 0.937-1.904 | 0.109 |
| TP53 mutation | 0.763 | 0.536-1.087 | 0.134 |
| Seven-lncRNA risk score (*N* = 503) | 2.537 | 2.067-3.114 | <0.001 |
| Age | 0.996 | 0.983-1.009 | 0.530 |
| Gender | 1.280 | 0.923-1.773 | 0.139 |
| BRAF mutation | 1.405 | 0.778-2.536 | 0.259 |
| Seven-lncRNA risk score (*N* = 487) | 2.402 | 1.974-2.923 | <0.001 |
| Age | 0.996 | 0.983-1.008 | 0.497 |
| Gender | 1.084 | 0.775-1.516 | 0.639 |
| CIMP | 0.505 | 0.305-0.837 | 0.008 |
| Seven-lncRNA risk score (*N* = 455) | 2.199 | 1.791-2.700 | <0.001 |
| Age | 0.997 | 0.983-1.011 | 0.669 |
| Gender | 1.351 | 0.936-1.948 | 0.108 |
| CIN status | 1.027 | 0.654-1.613 | 0.909 |
| Seven-lncRNA risk score (*N* = 557) | 2.322 | 1.964-2.746 | <0.001 |
| Age | 1.001 | 0.989-1.013 | 0.894 |
| Gender | 1.263 | 0.932-1.712 | 0.133 |
| Tumor location | 0.686 | 0.499-0.943 | 0.020 |
| Abbreviations: HR, hazard ratio; CI, conﬁdence interval; CIMP, CpG island methylator phenotype; CIN, chromosomal instability.  In Cox regression analyses, risk score was evaluated as continuous variables, all other variables were evaluated as category variables.  ^a^ *P* < 0.05 was considered statistically signiﬁcant in all analyses. | | | |

| **Table S4** Multivariable Cox regression analysis of the seven-lncRNA risk score and all eleven available clinical variables in GSE39582 data set (*N* = 249). | | | |
| --- | --- | --- | --- |
| **Variables** | **HR** | **95% CI of HR** | ***P* value^a^** |
| Seven-lncRNA risk score | 2.491 | 1.729-3.587 | <0.001 |
| Age | 1.002 | 0.983-1.021 | 0.860 |
| Gender | 1.656 | 0.999-2.744 | 0.050 |
| Stage | 2.004 | 1.393-2.882 | <0.001 |
| Adjuvant chemotherapy | 0.52 | 0.298-0.907 | 0.021 |
| KRAS mutation | 0.533 | 0.318-0.892 | 0.017 |
| MMR status | 2.258 | 0.794-6.418 | 0.127 |
| TP53 mutation | 0.76 | 0.456-1.267 | 0.292 |
| BRAF mutation | 0.251 | 0.043-1.479 | 0.127 |
| CIMP | 0.218 | 0.047-1.01 | 0.052 |
| CIN status | 0.538 | 0.27-1.07 | 0.077 |
| Tumor location | 0.893 | 0.535-1.49 | 0.665 |
| Abbreviations: HR, hazard ratio; CI, conﬁdence interval; MMR, mismatch repair; CIMP, CpG island methylator phenotype; CIN, chromosomal instability.  In Cox regression analyses, risk score was evaluated as continuous variables, all other variables were evaluated as category variables.  ^a^ *P* < 0.05 was considered statistically signiﬁcant in all analyses. | | | |
